# Manipulation of bicarbonate concentration in sperm capacitation media improvesin vitro fertilisation output in porcine species

**DOI:** 10.1186/s40104-019-0324-y

**Published:** 2019-03-11

**Authors:** Cristina Soriano-Úbeda, Jon Romero-Aguirregomezcorta, Carmen Matás, Pablo E. Visconti, Francisco A. García-Vázquez

**Affiliations:** 1 Department of Physiology, Faculty of Veterinary Science, International Excellence Campus for Higher Education and Research “Campus Mare Nostrum”, University of Murcia, 30100 Murcia, Spain; 2 Institute for Biomedical Research of Murcia (IMIB-Arrixaca), Murcia, Spain; 3 Department of Physiology, Faculty of Medicine and Nursing, University of the Basque Country UPV/EHU, Leioa, Bizkaia Spain; 4 Department of Veterinary and Animal Sciences, University of Massachusetts, Amherst, MA USA

**Keywords:** Adcy10, Boar, Monospermy, PKA

## Abstract

**Background:**

The in vivo concentration of bicarbonate (HCO_3_^−^), one of the essential sperm capacitating effectors, varies greatly in the different environments sperm go through from cauda epididymis to the fertilisation site. On the contrary, porcine in vitro sperm capacitation and fertilisation media usually contains a standard concentration of 25 mmol/L, and one of the main problems presented is the unacceptable high incidence of polyspermy. This work hypothesised that by modifying the HCO_3_^−^ concentration of the medium, the output of in vitro sperm capacitation and fertilisation could be increased.

**Results:**

Once exposed to the capacitation medium, the intracellular pH (pH_i_) of spermatozoa increased immediately even at low concentrations of HCO_3_^−^, but only extracellular concentrations of and above 15 mmol/L increased the substrates protein kinase A phosphorylation (pPKAs). Although with a significant delay, 15 mmol/L of HCO_3_^−^ stimulated sperm linear motility and increased other late events in capacitation such as tyrosine phosphorylation (Tyr-P) to levels similar to those obtained with 25 mmol/L. This information allowed the establishment of a new in vitro fertilisation (IVF) system based on the optimization of HCO_3_^−^ concentration to 15 mmol/L, which led to a 25.3% increment of the viable zygotes (8.6% in the standard system vs. 33.9%).

**Conclusions:**

Optimising HCO_3_^−^ concentrations allows for establishing an IVF method that significantly reduced porcine polyspermy and increased the production of viable zygotes. A concentration of 15 mmol/L of HCO_3_^−^ in the medium is sufficient to trigger the in vitro sperm capacitation and increase the fertilisation efficiency in porcine.

**Electronic supplementary material:**

The online version of this article (10.1186/s40104-019-0324-y) contains supplementary material, which is available to authorized users.

## Background

Fertilisation is considered a multifactorial process in which many factors are directly and/or indirectly involved. The success rate of this physiological process depends to a high degree on the ability of spermatozoa to undergo capacitation and penetrate the oocyte vestments [1]. In vivo sperm capacitation is a gradual event that is actively and progressively coordinated within succeeding segments of the female genital tract [2]. The changes occurring in spermatozoa during in vivo capacitation begin as soon as they are ejaculated as a result of, among other reasons, the bicarbonate (HCO_3_^−^) intake, subsequent activation of cAMP synthesis, intracellular pH alkalinisation (pH_i_), increase in intracellular Ca^2+^ concentration and motility activation (fast capacitation events) [3]. Once inside the female genital tract, other events occur in a sequential and parallel way for an extended period, such as hyperactivation, tyrosine phosphorylation (Tyr-P) and preparation to undergo acrosome reaction (AR) (slow capacitation events). Both fast and slow events depend on the HCO_3_^−^ and Ca^2+^ concentrations, but the slow ones also depend on the presence of cholesterol acceptors like albumin (reviewed by Visconti [3]). The inward transport of HCO_3_^−^ from the surrounding medium during capacitation involves electrogenic Na^+^/HCO_3_^−^ co-transporter (NBC) and Cl^−^/HCO_3_^−^ anion exchangers [4]. It starts with induction of the atypical soluble adenylyl cyclase Adcy10 aka sAC (from now on Adcy10), increase in cAMP levels and activation of protein kinase A (PKA) [5]. Numerous studies have indicated that PKA phosphorylates several substrates (pPKAs) which supposes the initial point either for fast or slow events of the capacitation [3]. In porcine sperm, previous studies suggest that phosphorylation of pPKAs rapidly increases in ~ 160 s through the action of HCO_3_^−^, after which the activity increases slowly to reach a maximum at 21 min [6]. The HCO_3_^−^ acts as a key capacitating agent and its importance in mediating changes in spermatozoa has been demonstrated in multiple studies in mice and humans. Knock-out mice that fail to express Adcy10 were infertile [7, 8] and females with defective HCO_3_^−^ secretion in the uterus produced low sperm capacitation and fertility [9].

After maturing as they pass through the epididymis, the spermatozoa are stored in the epididymal cauda in low HCO_3_^−^ conditions (~ 4 mmol/L [10]). During ejaculation, the spermatozoa come into contact with the seminal plasma, which contains higher HCO_3_^−^concentrations (~ 20 mmol/L) [11] but also decapacitating factors, mainly spermadhesins from accessory sex glands, that coat and stabilise the sperm surface to prevent premature capacitation [12]. Once in the female genital tract, sperm are subjected to different selection mechanisms that remove the immature or damaged ones. As they move forward in the uterus towards the oviduct, the spermatozoa have to swim through several environments increasingly rich in HCO_3_^−^ (~ 25 mmol/L [7]) and the decapacitating factors are gradually removed, enabling membrane destabilisation and capacitation. Inside the oviduct, they remain attached to the epithelium after the utero-tubal junction, in the caudal isthmus, establishing the sperm reservoir. Once ovulation occurs, the spermatozoa are gradually released from the sperm reservoir and swim towards the fertilisation site at the ampullary-isthmic junction [13]. However, many of the ovulation signals and mechanisms of gradual sperm release from the reservoir are still unknown, and, in vitro conditions are far from ideal for gradually exposing spermatozoa to capacitating conditions. Currently, most of the in vitro capacitation and fertilisation (IVF) methods, in porcine and other species, expose all the spermatozoa to a fixed HCO_3_^−^ concentration of 25 mmol/L at the same time [14–19], providing a static IVF system that little resembles the dynamic system of fluids to which gametes are exposed in vivo. Despite the development of chemically defined fertilisation media [15, 20, 21], different spermatozoa and oocytes co-incubation times [22], sperm capacitation methods [23] and the use of innovative IVF methods in straws or in devices in which both gametes are physically separated [24], polyspermy, one of the major problems in porcine IVF, persists. The microenvironment of in vitro capacitation and fertilisation currently used in laboratories is probably far from optimal for porcine species. The objective of this work was to study the effect of different concentrations of HCO_3_^−^ on sperm capacitation, mimicking as far as possible the increase that takes place in vivo. For this purpose, different HCO_3_^−^ concentrations were tested on PKAs activation, pH_i_ and sperm motility, and on several subsequent events downstream of PKA activation in the cascade of capacitation, such as Tyr-P and the AR. Finally, the in vitro fertility parameters were studied. The results of this study could increase our knowledge of sperm physiology and lead to an improvement of in vitro sperm capacitation and oocyte fertilisation output in porcine species.

## Material and methods

### Spermatozoa collection and preparation

Epididymes from mature Large White boars (*Sus scrofa*) were obtained from a slaughterhouse and transported to the laboratory within 30 min of slaughter. The cauda epididymis was dissected and the epididymal duct was isolated. A 24G BD Insyte™ catheter (381212, Becton Dickinson Infusion Therapy Systems, Inc., Sandy, Utah, USA) adapted to a syringe was inserted into the epididymal duct, and sperm samples were obtained by pressurised air through the deferens duct. Spermatozoa samples were incubated for up to 60 min (1, 5, 15, 30 and 60 min, depending on the experiment) in modified Tyrode’s albumin lactate pyruvate media (TALP) based on that described by Rath et al. [15], composed by 114.06 mmol/L NaCl, 3.20 mmol/L KCl, 0.50 mmol/L MgCl_2_•6H_2_O, 18.00 mmol/L sodium lactate, 0.35 mmol/L NaH_2_PO_4_•H_2_O, 5.00 mmol/L glucose, 2.00 mmol/L caffeine, 8.00 mmol/L calcium lactate•5H_2_O, 1 mg/mL polyvinyl alcohol, 0.17 mmol/L kanamycin and 0.003 mmol/L phenol red, 3 mg/mL bovine serum albumin (BSA), 1 mmol/L sodium pyruvate, but containing different HCO_3_^−^ concentrations: 0, 5, 15 or 25 mmol/L. Prior to sperm incubation, all media were adjusted to pH 7.4. The pH of the medium containing 0 mmol/L was adjusted using NaOH and HCl, the pH of the media containing 5, 15 and 25 mmol/L was adjusted in an incubator for nearly 3 h at 38.5 °C, saturated humidity and 1.0%, 3.0% or 5.0% of CO_2_ atmosphere in air, respectively, according to the Henderson-Hasselbalch equation. A group of spermatozoa incubated in phosphate buffer saline (PBS) was included as non-capacitating control (NCAP).

### Western blotting (WB)

Isolated proteins from 1 × 10^6^ spermatozoa samples were obtained as described by Navarrete et al. [25]. Briefly, after incubation for up to 60 min in TALP containing 0, 5, 15 or 25 mmol/L of HCO_3_^−^ or in NCAP medium, samples were centrifuged and washed in 500 μL PBS. The supernatant was removed, 10 μL Laemmli buffer [26] was added to the pellet, boiled for 5 min and centrifuged once more. Supernatant was supplemented with 5% β-mercaptoethanol, boiled for 3 min, loaded in SDS-PAGE gels (ref. 4561044, BioRad, Hercules, CA, USA) and electro-transferred to 0.45 μm PVDF membranes (Merk KGaA, Darmstadt, Germany). Membranes were first blocked with 5% BSA (A9647, Sigma-Aldrich®, Madrid, Spain) in TBS containing 0.1% Tween 20 (P1379, Sigma-Aldrich®, Madrid, Spain) (T-TBS) and then incubated with the primary polyclonal antibodies anti-phospho-PKA substrates (9624, Cell Signaling Technology, Beverly, USA, 1:2,000) and anti-β-actin HRP (β-act) (AB20272, Abcam PLC, Cambridge, UK, 1:5,000) or anti-β-tubulin (β-tub) (T0198, Sigma-Aldrich®, Madrid, Spain, 1:5,000). Secondary antibodies were diluted in T-TBS (1:10,000). For membranes stripping, a solution of 2% SDS, 0.74% β-mercaptoethanol, 62.5 mmol/L Tris and pH 6.5 was used at 60 °C for 20 min. Blots were visualised by chemiluminescence (Amersham Imager 600, GE Healthcare) using a Pierce® ECL 2 Western Blotting Substrate (80196, Lumigen Inc., Southfield, MI, USA). The relative amount of signal in each membrane was semi-quantified using the ImageQuant TL v8.1 software (GE Healthcare, Life Sciences, Buckinghamshire, UK).

### Spermatozoa intracellular pH (pH_i_)

The analysis of pH_i_ was carried out in spermatozoa (30 × 10^6^ cells/mL) loaded with 5 μmol/L of the pH-sensitive dye BCECF-AM (B1150, Sigma-Aldrich®, Madrid, Spain) for 30 min at 38.5 °C. After that, the samples were centrifuged at 700×*g* for 3 min to remove the excess of dye and resuspended in PBS without Ca^2+^ and Mg^2+^ and incubated again for 15 min at 38.5 °C for the de-esterification of the dye. Finally, the samples were centrifuged and resuspended in NCAP, 0 mmol/L, 5 mmol/L, 15 mmol/L and 25 mmol/L of HCO_3_^−^ for 1 and 60 min. The fluorescence was monitored using a spectrofluorometer (FP-6300, Jasco®, Cremella, Italy) every 2 s for a total time of 300 s. A calibration of the system was first performed using BCECF-AM stained and equilibrated spermatozoa at pH 6.0, 6.5, 7.0, 7.5 and 8.0 in the presence of 0.1% Triton X-100 by adjusting the pH with HCl and NaOH [27]. The emitted fluorescence ratio from the excitation at 490/440 nm was calculated and the regression line for extracellular pH (pH_e_) vs. the 490/440 nm ratio was obtained (Additional file 1). The pH_i_ of sperm cells was estimated from the regression line.

### Spermatozoa motility

Sperm motion assay was carried out using a computer-assisted spermatozoa motility analysis (CASA), the ISAS® system (PROISER R + D S.L., Valencia, Spain), connected to a negative phase-contrast microscope (magnification × 100; Leica DMR, Wetzlar, Germany) and a digital camera (Basler Vision, Ahrensburg, Germany). Spermatozoa motility was determined after 1, 15, 30 and 60 min of incubation in the different TALP media previously described. For this purpose, 4 μL of samples were placed on a warmed (38.5 °C) 20-μm SpermTrack® chamber (STP-21006, PROISER R + D S.L., Valencia, Spain) and motility parameters were determined into 3 different fields per sample and time of incubation: the percentage of total motile spermatozoa (Mot, %), motile progressive spermatozoa (MotPro, %), curvilinear velocity (VCL, μm/s), straight line velocity (VSL, μm/s), average path velocity (VAP, μm/s), linearity of the curvilinear trajectory (LIN, ratio of VSL/VCL, %), straightness (STR, ratio of VSL/VAP, %), wobble of the curvilinear trajectory (WOB, ratio of VAP/VCL, %), amplitude of lateral head displacement (ALH, μm) and beat cross-frequency (BCF, Hz). Moreover, the spermatozoa from each replicate were classified in 3 different clusters per experimental group and time of incubation to identify groups of spermatozoa with a high degree of similarity in their motility pattern: i) Cluster 1: spermatozoa with the poorest motility (lowest values in all CASA parameters); ii) Cluster 2: spermatozoa with the most linear trajectory (high values of VSL, LIN, STR and WOB); and iii) Cluster 3: spermatozoa with the most curvilinear trajectory (high values of VCL and ALH and low values of VSL, LIN and STR) (Additional file 2).

### Spermatozoa tyrosine phosphorylation (Tyr-P)

Indirect immunofluorescence (IIF) was performed as described by Luño et al. [28] to study sperm Tyr-P. Samples processing was carried out at 4 °C. After 60 min of incubation in TALP media containing different HCO_3_^−^ concentrations, sperm samples were first washed with PBS and centrifuged at 270×*g* for 10 min and then fixed in 2% p-formaldehyde/PBS for 60 min. The sample blocking was carried out overnight in a wet chamber in 2% (*w*/*v*) BSA in PBS. After that, samples were re-centrifuged at 270×*g* for 10 min, resuspended in PBS and spread on glass slides. The primary antibody used for incubation was anti-phosphotyrosine (4G10, Millipore, CA, USA, 1:300 in 0.1% BSA in PBS). The slides were washed with PBS and then incubated with the secondary antibody, fluorescein-conjugated goat anti-mouse (Bio-Rad Laboratories, Madrid, Spain, 1:400 in 0.1% BSA in PBS). Finally, samples were washed again with PBS and mounted with coverslips using a fluorescent medium (Dako, Carpinteria, CA, USA). Two-hundred spermatozoa per sample were analysed under an epifluorescence microscope (magnification × 400; Leica® DMR, USA). The Tyr-P location of sperm proteins was classified and grouped into 4 different categories according to the 4 patterns described by Luño et al. [28]: i) Pattern I (low capacitation), which included spermatozoa without fluorescence or with phosphorylated acrosome or tail or acrosome and tail; ii) Pattern II (medium capacitation), which included spermatozoa with fluorescence in the equatorial subsegment with or without the presence of signal in the flagellum; iii) Pattern III (high capacitation), which included spermatozoa with signal in the equatorial subsegment and acrosome area and with or without the presence of signal in the flagellum. A fourth pattern was established related to the motility activation which included those spermatozoa with a signal in the flagellum regardless of other locations (Pattern IV) (Additional file 3).

### In vitro fertilisation (IVF)

Porcine oocytes were isolated from ovaries obtained from 6- to 7-month-old animals at a local slaughterhouse and transported to the laboratory in saline solution at 38.5 °C. Once in the laboratory, the ovaries were washed in 0.04% cetrimide solution (*w*/*v*) and saline solution. Cumulus-oocytes complexes (COCs) were collected from antral follicles (3–6 mm diameter) and washed twice with Dulbecco’s PBS. Groups of 50 COCs with complete and dense cumulus oophorus were in vitro matured in 500 μL NCSU-37 medium based on that described by Petters and Wells [29] composed by 108.73 mmol/L NaCl, 25 mmol/L NaHCO_3_, 4.78 mmol/L KCl, 1.19 mmol/L KH_2_PO_4_, 1.19 mmol/L MgSO_4_•7H_2_O, 1.70 mmol/L CaCl_2_•2H_2_O, 5.55 mmol/L glucose, 1.00 mmol/L glutamine, 12.00 mmol/L sorbitol, 0.18 mmol/L penicillin and 0.07 mmol/L streptomycin, for 42 h, as previously described by Funahashi et al. [30]. After IVM, the COCs were gently pipetted to remove the cumulus. Groups of 50 oocytes were transferred to a 4-well multidish containing 500 μL per well of TALP media containing the different HCO_3_^−^ concentrations: 0, 5, 15 or 25 mmol/L described above. Each insemination dose comprised a pool of epididymal spermatozoa from 3 different boars. The spermatozoa were directly inseminated without being previously capacitated (monophasic IVF) or pre-incubated in capacitating media for 60 min with different HCO_3_^−^ concentrations and then inseminated (biphasic IVF), in both cases giving a final concentration of 5 × 10^4^ cells/mL. Biphasic IVF was established according to the different combinations of HCO_3_^−^ concentration in sperm pre-incubation and IVF, defining the experimental groups first with the concentration in pre-incubation and then in IVF: pre-incubation in 5 mmol/L and IVF in 15 mmol/L (5–15); 5–25; 15–15; 15–25; 25–25. After 18 h of co-culture, putative zygotes were fixed and IVF parameters were evaluated under an epifluorescence microscope at × 400 (Leica® DMR, USA) as previously described by Matás et al. [31]: the percentage of penetrated oocytes (Pen, %), percentage of monospermy of penetrated oocytes (Mon, %), number of spermatozoa penetrating each oocyte (SPZ/O), number of sperm bound to ZP (SPZ/ZP) and efficiency (%) which represents the final number of putative zygotes in each group per 100 penetrated oocytes.

## Statistical analysis

All statistical analyses were performed using IBM SPSS v.19 (SPSS Inc. Chicago, IL, USA) and data were analysed by one-way ANOVA. In the motility experiment, 2 types of analysis were carried out per field: i) data were analysed using the general linear repeated-measures procedure, and ii) data were grouped by a non-hierarchical k-means clustering for each time of incubation and a repeated-measures procedure was followed for each cluster. Three different clusters centers where established per motility parameter to classify each spermatozoa (Additional file 2). In both sets of analyses, the obtained results were compared by one-way ANOVA. Data are expressed as the mean ± standard error of the mean (SEM). Differences were considered statistically significant at *P* < 0.05, and when these analyses revealed a significant effect, values were compared using the least significant difference pairwise multiple comparison *post-hoc* test (Tukey). A statistical tendency was considered when *P*≥ 0.05 and ≤ 0.75.

## Results

### Effect of HCO_3_^−^ concentration on spermatozoa functionality

Among the ions existing along the route that spermatozoa must follow from the epididymis, HCO_3_^−^ has an important role in two main sperm functions: i) collaborating in the functional maturation of spermatozoa by affecting membrane dynamics and ii) buffering the environment [32–34]. Moreover, HCO_3_^−^ is a key factor in inducing sperm capacitation by direct stimulation of Adcy10 before triggering the cascade of fast and slow events of capacitation. Here, the effect of different HCO_3_^−^ concentrations on these capacitating events was investigated.

#### Spermatozoa protein kinase A substrates phosphorylation (pPKAs)

**Fig. 1:**
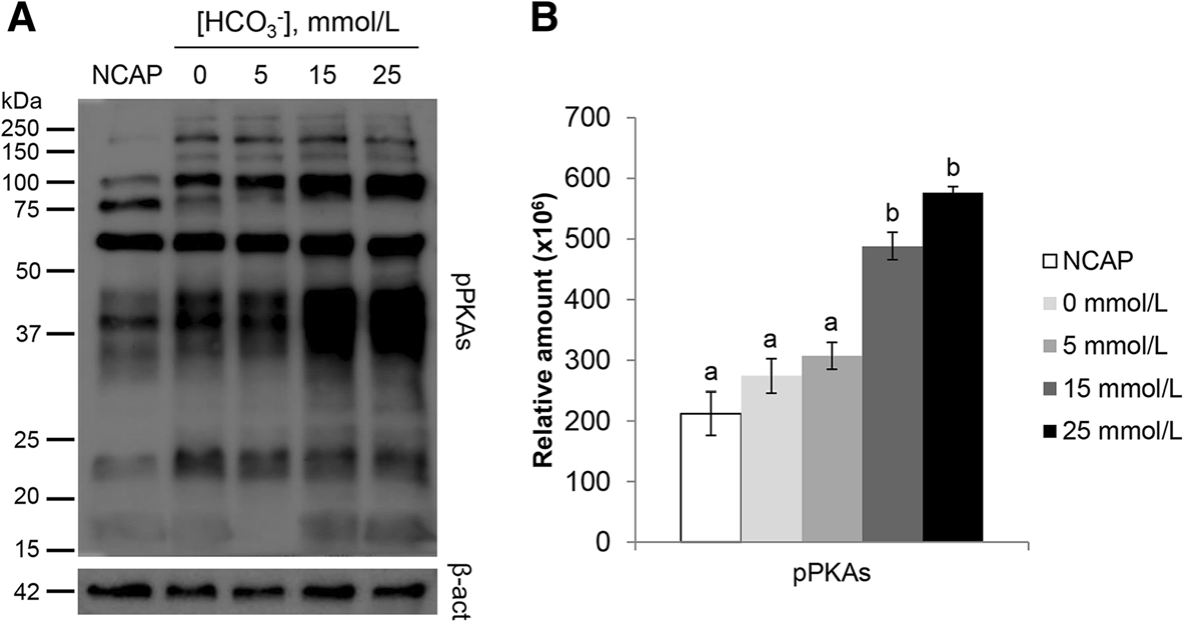
Effect of HCO_3_^−^ concentration on sperm protein kinase A substrates phosphorylation (pPKAs). **A)** pPKAs of sperm samples incubated for 60 min in capacitating medium (TALP) with different concentrations of HCO_3_^−^ (0 mmol/L, 5 mmol/L, 15 mmol/L and 25 mmol/L) and in PBS as non-capacitating control (NCAP) in 4 replicates. β-actin (β-act) was used as loading control. **B)** Relative amount of semi-quantified signal for blots. Results are shown as mean ± SEM. Different letters (a, b) indicate statistically significant differences (*P* < 0.05)

**Fig. 2:**
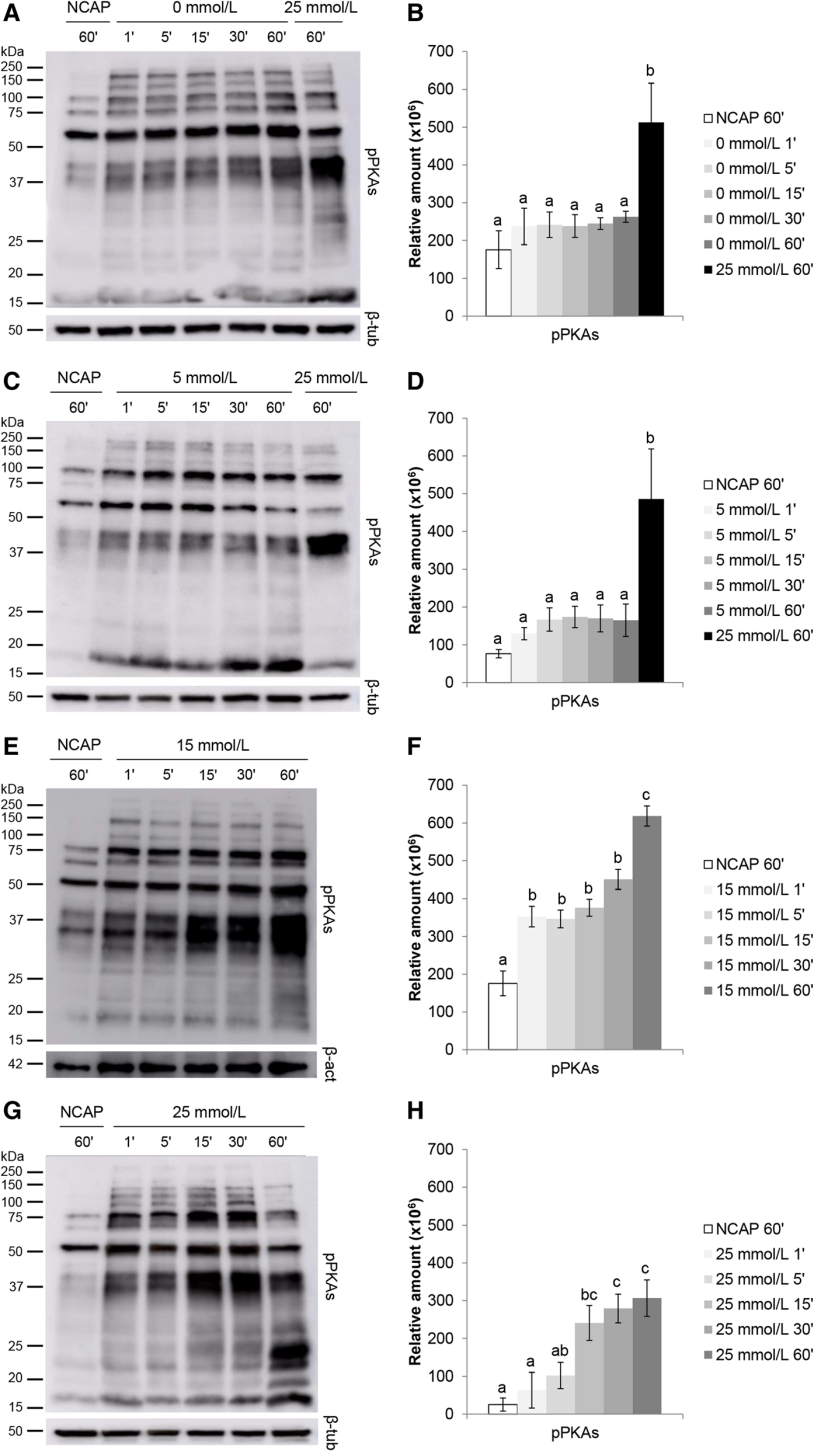
Kinetics of different HCO_3_^−^ concentrations on sperm protein kinase A substrates phosphorylation (pPKAs). Sperm were incubated for 1, 5, 15, 30 and 60 min in capacitating medium (TALP) containing 0 mmol/L (**A**), 5 mmol/L (**C**), 15 mmol/L (**E**) and 25 mmol/L (**G**) of HCO_3_^−^ (4 replicates). In each blot a lane of spermatozoa incubated in PBS was included as non-capacitating control (NCAP). In 0 mmol/L (**A**) and 5 mmol/L blots (**C**) a lane for spermatozoa incubated in 25 mmol/L for 60 min was included as positive control. β-actin (β-act) or β-tubulin (β-tub) was used as loading control. Relative amount of semi-quantified signal for 0 mmol/L, 5 mmol/L, 15 mmol/L and 25 mmol/L blots are shown in graphics **B**, **D**, **F** and **H**, respectively. Results are shown as mean ± SEM. Different letters (a-c) indicate statistically significant differences (*P* < 0.05) between groups

The effect of HCO_3_^−^ concentration on pPKAs after 60 min in NCAP, 0 mmol/L, 5 mmol/L, 15 mmol/L and 25 mmol/L groups are shown by WB in Fig. 1. The results showed that 15 mmol/L and 25 mmol/L produced a significantly higher degree of pPKAs compared with the rest of the groups (*P* < 0.05). Figure 1A showed that low concentrations of HCO_3_^−^ (0 and 5 mmol/L) produced low levels of pPKAs, similar to those produced in NCAP group. However, the addition of a higher HCO_3_^−^concentration (of and above 15 mmol/L) induced a significant increment in pPKAs. When comparing experimental replicates, 15 mmol/L and 25 mmol/L produced a significantly higher degree of pPKAs compared with the rest of the groups (*P* < 0.05) (Fig. 1B). Although it has been described that the pPKAs is time-dependent, as shown in Fig. 2, it is also concentration-dependent. When low HCO_3_^−^concentrations were used, like 0 or 5 mmol/L, the highest level of pPKAs was achieved since the beginning of incubation (Fig. 2A and C). On the other hand, when higher levels of HCO_3_^−^ were used, 15 and 25 mmol/L, pPKAs are progressively increasing (Fig. 2E and G), being faster as HCO_3_^−^ concentration is higher (25 mmol/L). Through semi-quantification it could be determined that inexistence or low concentrations of HCO_3_^−^ (0 or 5 mmol/L) could not provoke pPKAs similar to control (25 mmol/L) after 60 min of incubation (Fig. 2B and D). Higher concentrations of HCO_3_^−^ produce the highest pPKAs in a concentration-dependent way. A HCO_3_^−^ concentration of 15 mmol/L produced the highest pPKAs after 60 min of incubation (*P* < 0.05) and 25 mmol/L after 15 min (*P* < 0.05) (Fig. 2H).

#### Spermatozoa intracellular pH (pH_i_)

**Fig. 3:**
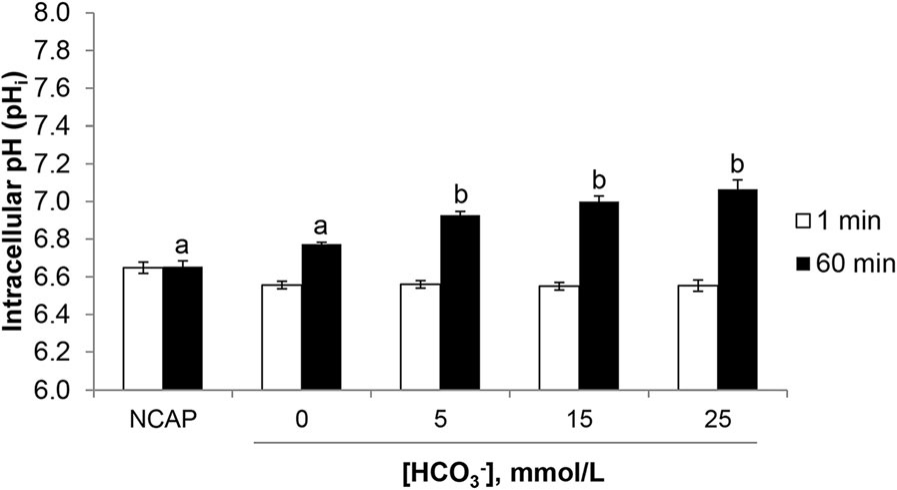
Effect of HCO_3_^−^ on boar epididymal sperm intracellular pH (pH_i_). Samples were loaded with 5 μmol/L of the pH-sensitive dye BCECF-AM for 30 min at 38.5 °C, centrifuged at 700×*g* for 3 min to remove the excess of dye and resuspended in PBS without Ca^2+^ and Mg^2+^ and incubated again for 15 min at 38.5 °C for the de-esterification of the dye. After that, sperm were incubated for 1 and 60 min in capacitating medium (TALP) containing different concentrations of HCO_3_^−^ (0 mmol/L, 5 mmol/L, 15 mmol/L and 25 mmol/L) and non-capacitating medium (NCAP) in 4 replicates. The fluorescence was monitored using a spectrofluorometer every 2 s for a total time of 300 s. The emitted fluorescence ratio from the excitation at 490/440 nm was calculated and the regression line for pH_e_ vs. the 490/440 nm ratio was obtained (Additional file 1). The pH_i_ of sperm cells was estimated from the regression line. Results are shown as mean ± SEM. Different letters (a, b) in the same time of incubation indicate statistically significant differences (*P* < 0.05)

Adcy10/cAMP activation and further cascade events (including PKAs phosphorylation) are boosted by the intracellular alkalinisation of spermatozoa associated with the intake of external HCO_3_^−^, but the relation with the specific HCO_3_^−^ concentration in capacitating conditions is still unclear. The pH_i_ of the spermatozoa exposed to NCAP or 0 mmol/L, 5 mmol/L, 15 mmol/L and 25 mmol/L of HCO_3_^−^ is shown in Fig. 3. At 1 min of incubation, there were no differences in the pH_i_ between groups (NCAP: 6.65 ± 0.03, 0 mmol/L: 6.56 ± 0.02, 5 mmol/L: 6.56 ± 0.02, 15 mmol/L: 6.55 ± 0.02, 25 mmol/L: 6.55 ± 0.03, *P* > 0.05). After 60 min of incubation, the pH_i_ of spermatozoa increased in all groups, but only those that contained HCO_3_^−^ were statistically higher than NCAP and 0 mmol/L (NCAP: 6.65 ± 0.03, 0 mmol/L: 6.77 ± 0.01, 5 mmol/L: 6.93 ± 0.02, 15 mmol/L: 7.00 ± 0.03, 25 mmol/L: 7.06 ± 0.05, *P* < 0.05).

#### Spermatozoa motility

**Fig. 4:**
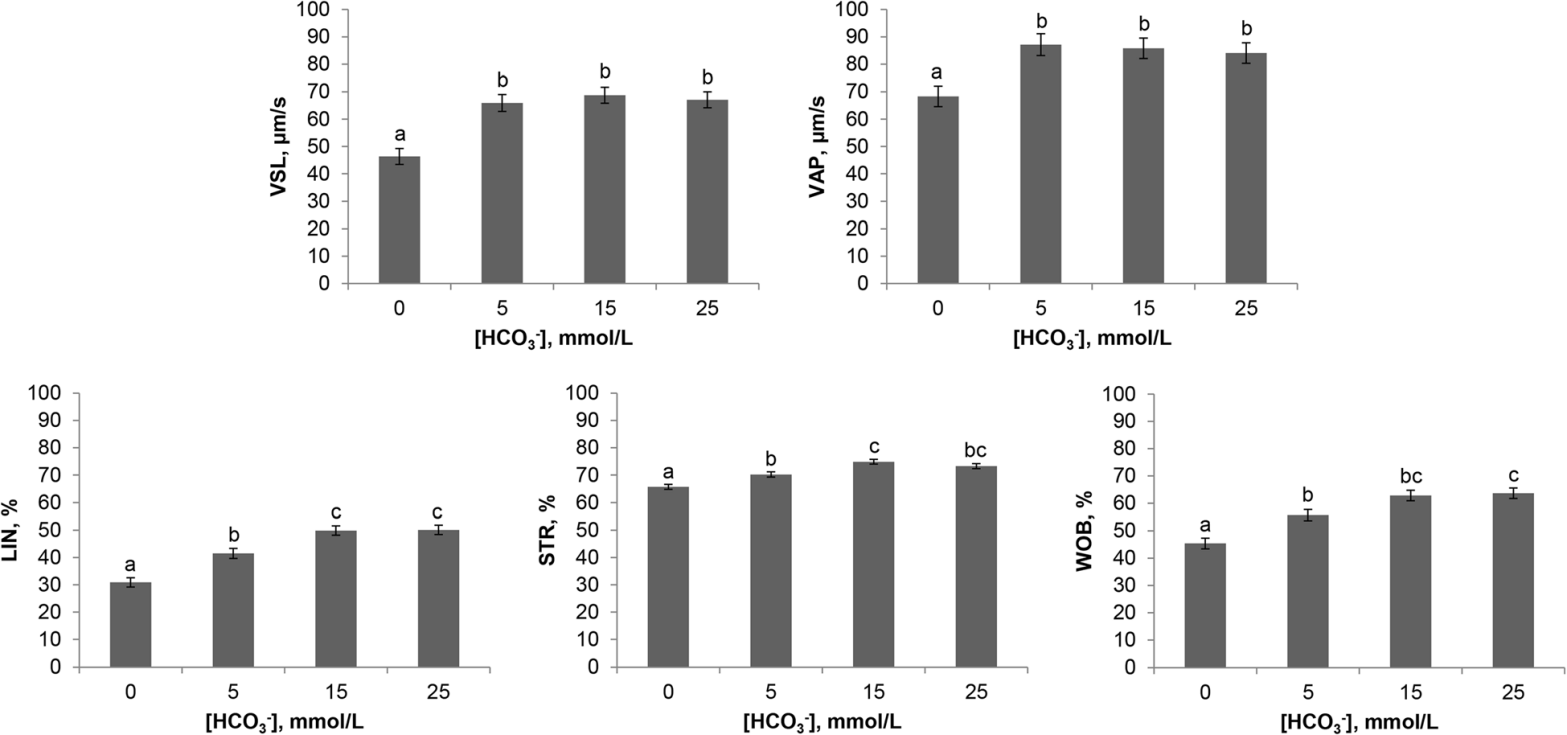
Effect of HCO_3_^−^ on epididymal sperm motility in capacitating conditions. Boar epididymal spermatozoa were incubated in capacitating medium (TALP) containing different concentrations of HCO_3_^−^ (0 mmol/L, 5 mmol/L, 15 mmol/L and 25 mmol/L) and CASA parameters were determined at 1, 15, 30 and 60 min of incubation in 3 replicates. VSL, μm/s: straight-line velocity; VAP, μm/s: average path velocity; LIN, %: linearity of the curvilinear trajectory (VSL/VCL); STR, %: straightness (VSL/VAP); WOB, %: wobble (VAP/VCL). The data per field were analysed by the general linear repeated-measures model and a Tukey test. Results expressed as mean ± SEM. Different letters (a-c) for graph bars indicate statistical differences (*P* < 0.05)

**Fig. 5:**
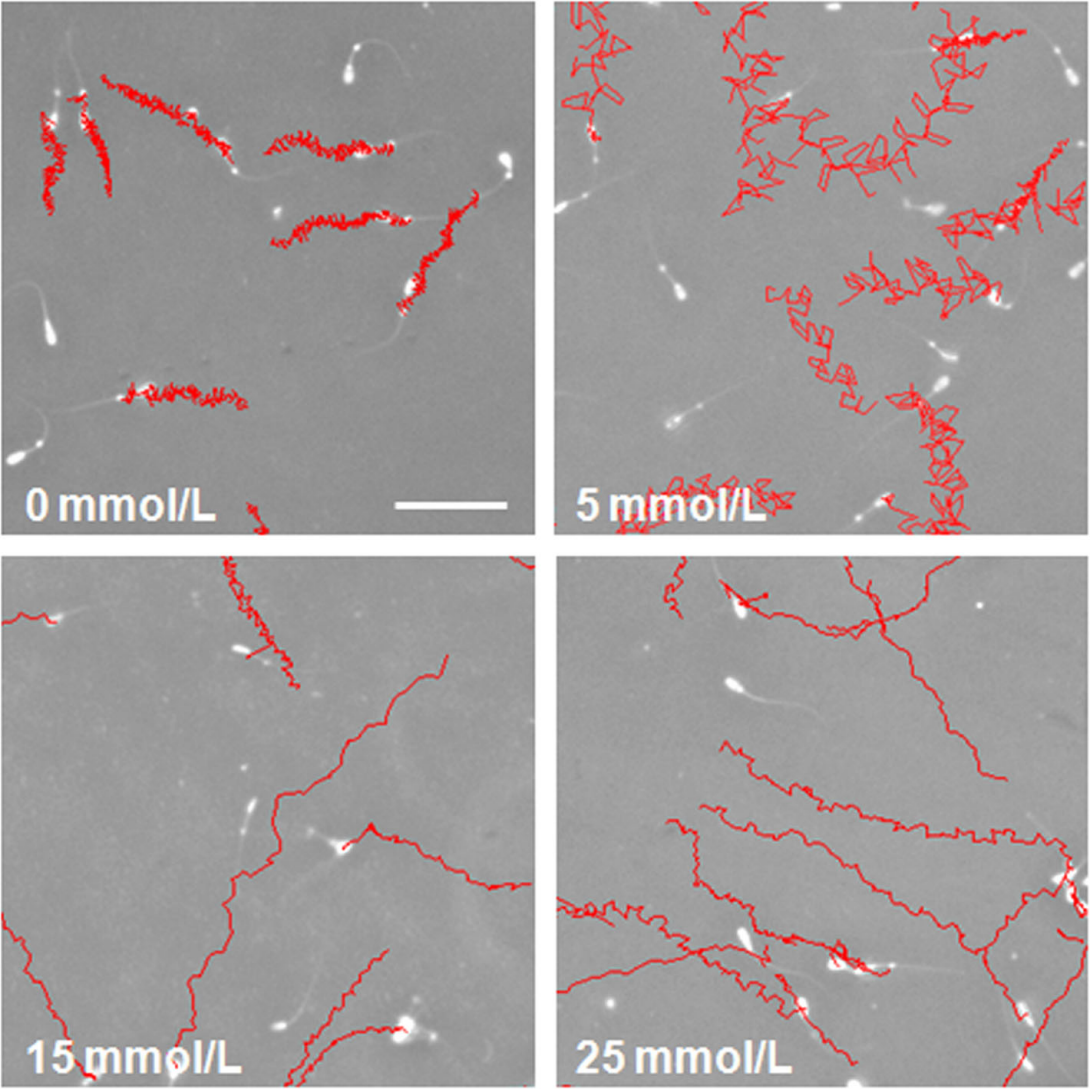
Comparison of epididymal sperm trajectories in capacitating conditions using different HCO_3_^−^ concentrations. Boar epididymal spermatozoa were incubated in capacitating medium (TALP) containing different concentrations of HCO_3_^−^ (0 mmol/L, 5 mmol/L, 15 mmol/L and 25 mmol/L) for 60 min. Sequences of 2 s were obtained by CASA (50 frames/s)

**Fig. 6:**
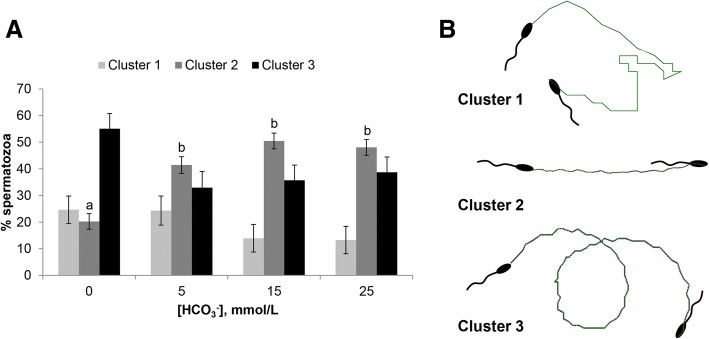
Clustering analysis of epididymal sperm trajectory in capacitating conditions with different HCO_3_^−^ concentrations. **A)** Boar epididymal spermatozoa were incubated in capacitating medium (TALP) containing different concentrations of HCO_3_^−^ (0 mmol/L, 5 mmol/L, 15 mmol/L and 25 mmol/L) and CASA parameters were determined at 1, 15, 30 and 60 min of incubation in 3 replicates. Clustering of spermatozoa was carried out per field. Cluster 1: lowest values in all CASA parameters (poorest motility). Cluster 2: high values of VSL, LIN, STR and WOB (the most linear trajectory). Cluster 3: high values of VCL and ALH and low of VSL, LIN and STR (the most curvilinear trajectory). Data were analysed by one-way ANOVA and a Tukey test. Results expressed as mean ± SEM. Different letters (a, b) in the different experimental groups (0 mmol/L, 5 mmol/L, 15 mmol/L and 25 mmol/L) of the same cluster indicate statistically significant differences (*P* < 0.05). **B)** Illustrations are representative of the 3 clusters defined

One of the first events related with Adcy10/PKA pathway activation by HCO_3_^−^ is sperm motility stimulation. Thus, the effect of HCO_3_^−^ on sperm motility and trajectory was studied in capacitating conditions in 0 mmol/L, 5 mmol/L, 15 mmol/L and 25 mmol/L (Figs. 4, 5 and 6). The results showed that the HCO_3_^−^ concentration did not affect Mot (ranged from 84.9% to 92.9%), MotPro (from 83.2% to 92.4%) or VCL (from 138.2 to 165.0 μm/s) but stimulated straight and average path velocities (VSL and VAP, *P* < 0.05) (Fig. 4). A HCO_3_^−^ concentration of 15 mmol/L produced significantly higher LIN, STR and WOB than 0 mmol/L and 5 mmol/L but 25 mmol/L did not induce any extra improvement (Fig. 4). There were no statistical differences in ALH and BCF between groups. A visual representation of the effect of HCO_3_^−^ on the trajectories of spermatozoa is shown in Fig. 5. An increasingly straight trajectory was directly proportional to the HCO_3_^−^ concentration in the medium. Moreover, a cluster analysis revealed that the proportion of spermatozoa with poorer motility (cluster 1) was similar between groups, regardless of the HCO_3_^−^ concentration and length of incubation (Fig. 6). The proportion of spermatozoa with the most rectilinear trajectory (cluster 2) was significantly higher (*P* < 0.05) in the 5 mmol/L, 15 mmol/L and 25 mmol/L groups than in 0 mmol/L. In addition, and despite a certain tendency (*P* = 0.051) to decrease in the presence of HCO_3_^−^, there were no statistical differences in the proportion of spermatozoa with the most curvilinear trajectory (cluster 3) between groups.

#### Spermatozoa tyrosine phosphorylation (Tyr-P)

**Fig. 7:**
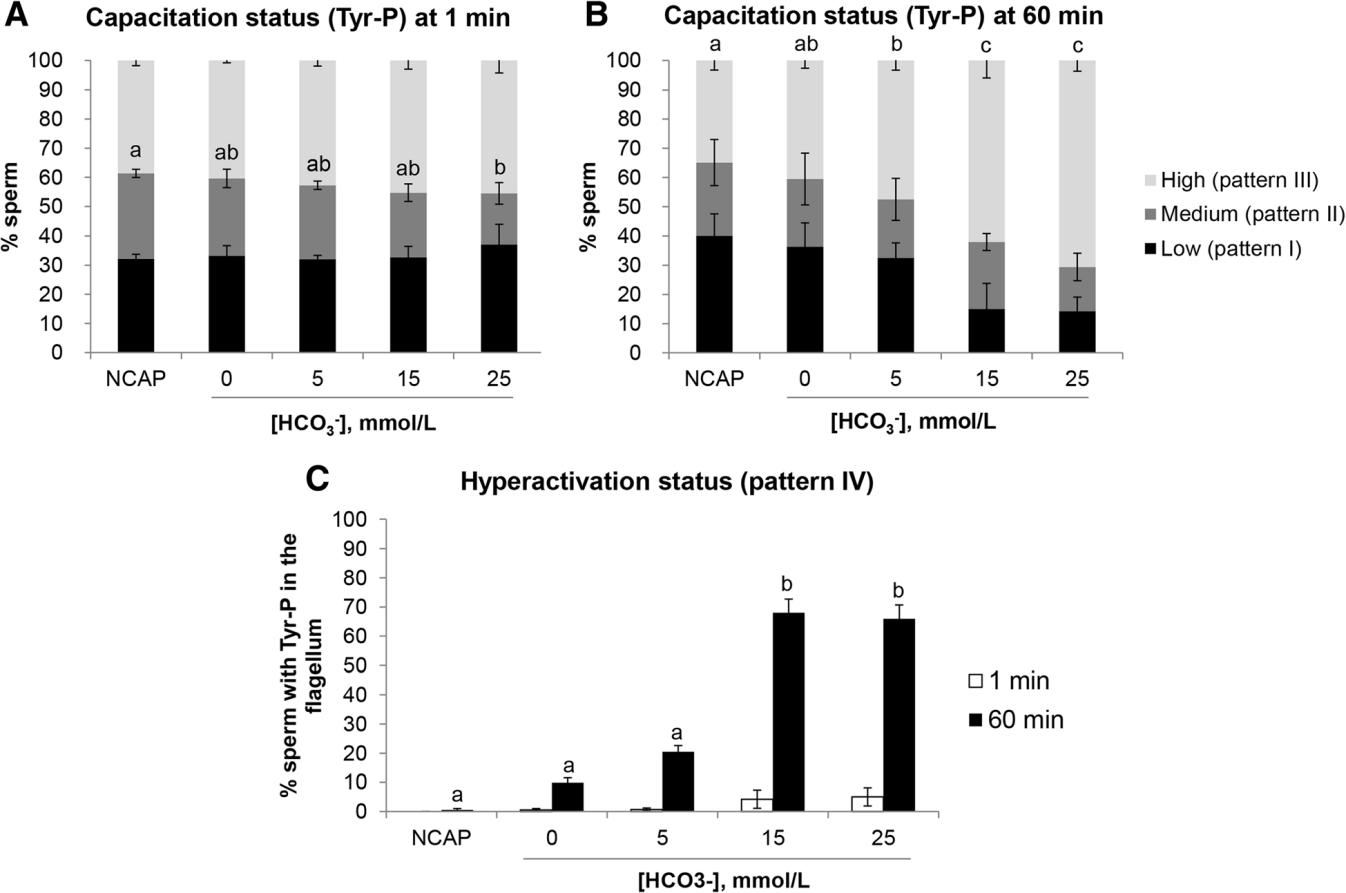
Immunolocation of protein tyrosine phosphorylation (Tyr-P) on boar epididymal sperm subjected to different bicarbonate concentrations. Sperm were incubated for 1 and 60 min in capacitating medium (TALP) containing different concentrations of HCO_3_^−^ (0 mmol/L, 5 mmol/L, 15 mmol/L and 25 mmol/L) and non-capacitating medium (NCAP) in 4 replicates. **A)** and **B)** Capacitation status at 1 and 60 min of incubation, respectively: percentage of sperm with low capacitation status (pattern I: non-phosphorylated or head- and/or flagellum-phosphorylated spermatozoa), medium capacitation status (pattern II: equatorial segment or equatorial segment and flagellum phosphorylated) or high capacitation status (pattern III: equatorial segment and head and/or flagellum phosphorylated). **C)** Pattern IV: flagellum phosphorylation regardless of phosphorylation in other locations at 1 and 60 min of incubation. Results are shown as mean ± SEM. Different letters (a-c) for the same time of incubation and pattern indicate statistically significant differences (*P* < 0.05)

The pH_i_ seems to favour the cascade of slow and fast events in spermatozoa, including the activation of tyrosine kinases and the subsequent Tyr-P of proteins. In fact, the capacitation status was described to be strongly correlated with Tyr-P [5] as a direct consequence of the action of HCO_3_^−^, whose omission not only inhibits Tyr-P [5], but also diminishes the ability of spermatozoa to bind to ZP [35]. This slow capacitation response must be preceded by lipid scrambling and cholesterol removal by a cholesterol acceptor, normally albumin. Different compartments have been described as undergoing a specific sequence of phosphorylation events during both capacitation and binding to ZP [36]. The present experiment tried to evaluate the effect of HCO_3_^−^ concentration on Tyr-P immunolocation in epididymal porcine spermatozoa (Fig. 7). At 1 min of incubation, all the experimental groups had the same proportion of spermatozoa with patterns I and III (low and high capacitation status, respectively). The 25 mmol/L concentration had a significantly reduced proportion of spermatozoa with pattern II (medium capacitation status) (17.4 ± 3.7%) compared with NCAP (29.2 ± 1.4%) (Fig. 7A). At 60 min of incubation, the proportion of pattern I and II (low and medium capacitation status, respectively) did not show any statistical difference between the media used (*P* > 0.05). However, the proportion of pattern III (high capacitation status) was significantly higher in 15 mmol/L and 25 mmol/L (45.2 ± 3.0% and 45.5 ± 4.3%, respectively; *P* < 0.05) (Fig. 7B). As regards pattern IV, 15 mmol/L and 25 mmol/L produced the highest level of Tyr-P in the spermatozoa tail, which differed significantly from the rest of the groups (68.0 ± 4.7% and 65.9 ± 4.8%, respectively; *P* < 0.05) (Fig. 7C). Taken together, these results showed that a 15 mmol/L concentration of HCO_3_^−^ in the capacitation medium is sufficient to attain a high level of Tyr-P and probably motility activation.

### Effect of HCO_3_^−^ concentration on IVF

As described above, the concentration of HCO_3_^−^ affects sperm functionality and modulates sperm capacitation, a regulatory event that prepares spermatozoa for fertilisation prior to encountering the oocyte. Based on this, the present study tried to determine the fertility of spermatozoa and the interaction between gametes in the same conditions.

#### Monophasic IVF

The IVF output for penetration, monospermy, SPZ/O, SPZ/ZP and efficiency are shown in Table 1A. In the absence of HCO_3_^−^ (0 mmol/L) no oocyte was penetrated, which confirms that HCO_3_^−^ is an indispensable component of the IVF medium for the spermatozoa to bind to ZP and to penetrate the oocyte. When 5 mmol/L was used, all the studied parameters were significantly lower than when 15 mmol/L or 25 mmol/L was used. The 25 mmol/L concentration had a lower efficiency (8.6 ± 1.7%) than 15 mmol/L because, although penetration was high, monospermy was low. The use of 15 mmol/L significantly increased the efficiency of the technique to 26.2 ± 3.1%, with a lower penetration but a higher monospermy (*P* < 0.05) than at 25 mmol/L. In light of these results, it is clear that sperm fertility was affected by the mere presence of HCO_3_^−^ and also its concentration. This study then turned to the task of identifying an IVF method that increased the efficiency of obtaining potentially viable zygotes by optimizing the HCO_3_^−^ concentration to which gametes are gradually exposed.

**Table 1:** Effect of HCO_3_^−^ concentration on in vitro sperm fertility. **A**) Monophasic IVF: performed in TALP medium containing different HCO_3_^−^ concentrations (0 mmol/L, 5 mmol/L, 15 mmol/L and 25 mmol/L) for 18 h of co-culture at 38.5 °C. **B**) Biphasic IVF (Sperm pre-incubation + Monophasic IVF): sperm were pre-incubated in TALP for 60 min in 5 mmol/L, 15 mmol/L or 25 mmol/L and then IVF was performed in TALP medium with the same or higher HCO_3_^−^ concentration for 18 h of co-culture at 38.5 °C

A							
[HCO_3_^−^], mmol/L IVF		*n*	Pen, %	Mon, %	SPZ/O	SPZ/ZP	Efficiency, %
0		216	0^a^	–	–	0.3 ± 0.1^a^	0^a^
5		204	21.6 ± 2.9^b^	61.4 ± 7.4^a^	1.7 ± 0.2^a^	2.8 ± 0.2^b^	13.2 ± 2.4^b^
15		206	79.6 ± 2.8^c^	32.9 ± 3.7^b^	4.0 ± 0.3^b^	4.9 ± 0.5^c^	26.2 ± 3.1^c^
25		280	97.5 ± 0.1^d^	8.8 ± 1.7^c^	6.6 ± 0.3^c^	8.8 ± 0.5^d^	8.6 ± 1.7^b^
B							
[HCO_3_^−^], mmol/L		*n*	Pen, %	Mon, %	SPZ/O	SPZ/ZP	Efficiency, %
Sperm pre-incubation	IVF						
5	15	171	46.8 ± 3.8^a^	36.2 ± 5.4^ab^	3.0 ± 0.2^ab^	3.4 ± 0.2^a^	17.0 ± 2.9^a^
5	25	172	54.1 ± 3.8^a^	21.5 ± 4.3^a^	4.4 ± 0.3^c^	4.1 ± 0.3^abc^	11.6 ± 2.4^a^
15	15	165	69.7 ± 3.6^b^	48.7 ± 4.7^b^	2.4 ± 0.2^b^	3.6 ± 0.2^ab^	33.9 ± 3.7^b^
15	25	164	76.8 ± 3.3^bc^	25.4 ± 3.9^a^	3.6 ± 0.2^ac^	4.5 ± 0.2^bc^	19.5 ± 3.1^a^
25	25	171	86.6 ± 2.6^c^	24.3 ± 3.5^a^	3.4 ± 0.2^a^	4.8 ± 0.3^c^	21.0 ± 3.1^a^

Results are expressed as mean ± SEM. Different superscripts (^a-d^) in the same column indicate significant differences (*P* < 0.05)

P*en, %* penetration percentage of total oocytes used, *Mon, %* monospermy percentage calculated from penetrated oocytes, *SPZ/O* number of spermatozoa penetrating each oocyte, *SPZ/ZP* number of sperm bound to ZP, *Efficiency, %* final number of putative zygotes (monospermic) in each group per 100 penetrated oocyte

#### Biphasic IVF (spermatozoa pre-incubation + monophasic IVF)

The pre-incubation of sperm in a capacitating medium affected all the parameters studied (Table 1B). Penetration increased proportionally to the concentration of HCO_3_^−^ in sperm pre-incubation, although monospermy was more closely related to the HCO_3_^−^ concentration in the IVF medium, reaching between 21.5% and 25.4% when IVF was carried out at 25 mmol/L and between 36.2% and 48.7% when it was carried out at 15 mmol/L. Thus, the higher efficiency was achieved when both pre-incubation and IVF was performed in 15 mmol/L (33.9 ± 3.7%, *P* < 0.05).

## Discussion

Spermatozoa maturation in the epididymis comprises sequential modifications that have been demonstrated to be essential for the acquisition of motility and fertility [37]. Once in the epididymal cauda, where the HCO_3_^−^ concentration is minimal (~ 4 mmol/L), matured spermatozoa remain in a quiescent state. After ejaculation, spermatozoa are activated and changes in the sperm membrane begin as they progress through the uterus, where they find increasing concentrations of HCO_3_^−^. Subsequently, sperm capacitation is completed in the female isthmus and spermatozoa are again progressively exposed to increasing amounts of the capacitating effector HCO_3_^−^ from the isthmus to ampulla [38]. In the present study we have elucidated how epididymal spermatozoa respond to different HCO_3_^−^ concentrations during capacitation and in vitro fertilisation.

This study was carried out using mature spermatozoa that had never been in contact with high concentrations of HCO_3_^−^ or with decapacitant agents from accessory sex glands. Epididymal spermatozoa were incubated in a capacitating medium containing 0, 5, 15 or 25 mmol/L of HCO_3_^−^ and in non-capacitating medium. The results showed that the highest degree of PKAs activation was achieved by incubation in a capacitating medium containing 15 mmol/L of HCO_3_^−^ or above, although the 25 mmol/L concentration did not perform any better than 15 mmol/L. In spite of the presence of other capacitating agents such as calcium and albumin, concentrations lower than 15 mmol/L (for example the 5 mmol/L, which is similar to that existing in the epididymis) produced a low pPKAs, similar to that produced in the absence of HCO_3_^−^ (0 mmol/L), quantitatively matching the NCAP conditions which lack other capacitation effectors as albumin and calcium. This fact reflects that not only HCO_3_^−^ is necessary to initiate the capacitation cascade but also albumin and calcium, as has been previously described. It is known that defective HCO_3_^−^ secretion into the uterus may be related to impaired sperm capacitation and a low female fertility [9]. Therefore, a 15 mmol/L concentration of HCO_3_^−^ is postulated as being sufficient to achieve a high degree of PKAs activation that is essential in the sperm capacitation process. The differences in the results obtained with different HCO_3_^−^ concentrations were not dependent on the viability of the cells analysed (Additional file 4). Therefore, although this study showed the dose-dependent effect of HCO_3_^−^ on sperm capacitation, above 15 mmol/L there was no extra effect. However, the kinetic of PKAs activation allowed us to understand that the required time to activate PKAs does depend on HCO_3_^−^ concentration, which was rapidly activated at 1 min of incubation at concentrations of 15 and 25 mmol/L. These results agree with a previous study [6]. Furthermore, PKAs was highly phosphorylated after 30 min of incubation with 25 mmol/L and after 60 min with 15 mmol/L, demonstrating that a concentration of 15 mmol/L can produce a high degree of pPKAs, although it takes longer. The absence (0 mmol/L) or low concentrations of HCO_3_^−^ (5 mmol/L), similar to that existing in the cauda epididymis, did not enable PKAs activation even after 60 min of incubation, which shows that not only is the presence of HCO_3_^−^ essential in this pathway but also a minimum concentration is required. HCO_3_^−^ is a key activator of a cascade of events in capacitation and a minimum concentration and incubation time is necessary to trigger the capacitation process and produce a high pPKAs, despite the presence of other capacitating agents like calcium and albumin. 15 mmol/L of HCO_3_^−^ seems to be a sufficient concentration to allow epididymal sperm capacitation. Nevertheless, the importance of HCO_3_^−^ not only lies in the activation of PKAs but also the fact that it produces another series of events essential for sperm physiology. The increase in intracellular pH conditions, a phenomenon during capacitation (reviewed by Nishigaki et al. [39]) and the Adcy10/cAMP/PKA pathway are not an exception.

Epididymal spermatozoa in the cauda epididymis remain in a quiescent state favoured by low HCO_3_^−^ content and acidic pH [10]. The present study showed that, in the absence of HCO_3_^−^ and the presence of other capacitating agents like calcium and albumin, the pH_i_ remained at 6.7–6.8. In the presence of HCO_3_^−^, the pH_i_ increased 0.3–0.4 units, confirming that the increase depends exclusively on HCO_3_^−^. This increase of around 0.3–0.4 units in pH_i_ may seem small, but, it has been demonstrated that an increase of 0.2 units of pH may modify enzymatic activity more than 20-fold [40]. It is known that the activity of intra- and extra-cellular enzymes taking part in the cellular metabolism is pH-sensitive.

The effect of HCO_3_^−^ has been widely studied and there is evidence that extracellular HCO_3_^−^ influences the motility of epididymal spermatozoa of several species [10, 41, 42]. In the present study, HCO_3_^−^ concentration did not affect the proportion of motile spermatozoa during incubation in a capacitating medium, or their progressivity, but did affect parameters related to sperm trajectory. Holt and Harrison [43] showed in ejaculated, preserved and selected spermatozoa by Percoll gradient that the straight and average velocities, straightness and linearity significantly increased with the addition of HCO_3_^−^ to the incubation medium. The results of the present study presented evidence that HCO_3_^−^ activates motility in epididymal spermatozoa and produces a change in their trajectory which becomes more linear (see Fig. 5) and rapid. These data suggest that HCO_3_^−^ is not only important for motility activation in a balanced salt solution but also that the type of movement produced is concentration-dependent. A concentration of 15 mmol/L HCO_3_^−^ during sperm capacitation produced a more linear movement, which has previously been related with the state of capacitation in boar [43, 44].

The HCO_3_^−^ induction of an increase in LIN in porcine is controversial, since it has been previously reported by Satake et al. [45] using 15 mmol/L HCO_3_^−^, but it is in clear contrast to other studies [46] in which hyperactivation of ejaculated preserved spermatozoa was related to a decreased LIN. However, sperm motility in capacitating conditions seems to be species-specific, for instance in rodents as rat and hamster the hyperactivated motility is characterised by a decreased LIN [47, 48]. In our study, HCO_3_^−^ concentrations lower than 15 mmol/L produced a more curvilinear trajectory, while higher concentrations (25 mmol/L) did not produce any extra improvement over the value obtained at 15 mmol/L. However, each individual spermatozoon responds differently to HCO_3_^−^ [45] and the clustering analysis identified 3 different sperm subpopulations according to their trajectory in response to HCO_3_^−^. In this case, the proportion of spermatozoa with more linear trajectory was higher in the presence of HCO_3_^−^. While a concentration of HCO_3_^−^ from 15 mmol/L stimulated sperm motility and produced a change in the type of trajectory, no additional effect was obtained with 25 mmol/L. The role of HCO_3_^−^ in sperm capacitation is not only restricted to the immediate effects on spermatozoa (increased pH_i_, activation of motility and pPKAs) but is also important in slow capacitation events such as the increase in Tyr-P and AR. In vitro sperm capacitation is accompanied by a time-dependent increase in the Tyr-P of a subset of proteins, as one of the final steps of the PKA-dependent cascade of capacitation [5]. Sperm incubated in a medium lacking HCO_3_^−^ fails to undergo capacitation, but the process can be recovered by adding HCO_3_^−^ in a concentration-dependent way [5]. The present study showed that a concentration of 15 mmol/L is sufficient to produce an intracellular alkalinisation and a high degree of Tyr-P in spermatozoa but no extra effect was observed when 25 mmol/L was used. Something similar occurred with Tyr-P in the flagellum of spermatozoa. A high degree of pattern IV was observed at 15 mmol/L and above, although no significantly improvement was observed in 25 mmol/L. Overall, this study agrees with those of other authors [49] who showed that HCO_3_^−^ is required to activate Adcy10, which increases intracellular cAMP, promotes PKA activity and subsequent Tyr-P. The above-mentioned observations concerning the role of HCO_3_^−^ concentration on sperm functionality have shown the modulating power of HCO_3_^−^ on sperm physiology in capacitating conditions. However, whether this modulating effect influences the sperm fertilising ability during IVF has not been elucidated. In this study, the effect of HCO_3_^−^ concentration on sperm fertility was investigated (Table 1A). We hypothesised that varying the HCO_3_^−^ concentration in the porcine sperm capacitation process regulate their functionality and probably affect IVF output. Indeed, IVF was directly and strongly affected by the HCO_3_^−^ concentration in the medium. The importance of HCO_3_^−^ in the fertilisation process was confirmed by the fact that no oocyte was penetrated in the absence of HCO_3_^−^ (0 mmol/L). This agrees with Suzuki et al. [50], who described how the absence of HCO_3_^−^ in fertilisation media makes oocyte fertilisation impossible. In our study, we found that HCO_3_^−^ exerts a concentration-dependent impact on fertilisation as penetration increased with increasing HCO_3_^−^ concentrations. In addition, the presence of HCO_3_^−^ also upregulated the SPZ/O resulting in monospermy reduction.

The high incidence of polyspermy in porcine has been widely related with an unacceptably low efficiency of IVF [51]. Hunter and Nichol [52] hypothesised that the incidence of polyspermy is a direct consequence of the number of capacitated spermatozoa in the immediate vicinity of ovulated porcine oocytes in vivo. Later, Abeydeera and Day [21] also described this correlation under in vitro conditions. Results from this manuscript suggest that the effect on IVF of a specific HCO_3_^−^ concentration is related with the timing of the capacitation events and their influence in monospermic fertilisation success. At the molecular level, our results indicate that 5 mmol/L did not activate PKAs while 25 mmol/L produce faster phosphorylation compared to 15 mmol/L. The slower PKAs activation of 15 mmol/L could be responsible for the lower penetration and the higher monospermy than observed with 25 mmol/L in the fertilisation experiments. Moreover, Funahashi [53] described that one of the strategies to reduce polyspermy in porcine is to induce full AR in partially reacted spermatozoa. However, the results of the AR assay (Additional file 5) showed that polyspermy is not related with the proportion of acrosome-reacted spermatozoa surrounding the egg. Concentrations of 15 and 25 mmol/L HCO_3_^−^ in the capacitating media produce the same proportion of acrosome-reacted sperm after 60 min of incubation.

In addition to its effects in sperm penetration and monospermy parameters, HCO_3_^−^ had a determining action in the primary recognition between gametes, since practically no spermatozoon was able to bind to ZP in the absence of HCO_3_^−^. As Flesch et al. [54] suggested, it is probable that the absence of HCO_3_^−^ hindered certain capacitation events that resulted in reduced binding affinity of spermatozoa for ZP. One important question is the extent by which the influence of HCO_3_^−^ in IVF is exerted on spermatozoa during capacitation or on gamete co-incubation during the fertilisation process. After sperm pre-incubation with 15 mmol/L, which produced a moderate penetration, when IVF was also carried out at 15 mmol/L, monospermy was significantly higher and the SPZ/O lower than in the rest of groups, which resulted in a higher final efficiency (33.9%). What it is clear is that oocyte penetration directly increased with the HCO_3_^−^ concentration in the sperm pre-incubation medium suggesting that the action of HCO_3_^−^ is exerted more on spermatozoa than on oocytes. Very few studies have described the effect of HCO_3_^−^ on in vitro matured oocytes, but Suzuki et al. [50] suggested that, although HCO_3_^−^ was apparently affecting gamete interaction, it is highly probable that HCO_3_^−^ affects fertilisation through its effect on spermatozoa rather than through any effect on the oocytes.

## Conclusion

Altogether our results suggest that 15 mmol/L HCO_3_^−^ is sufficient for IVF media because it produces a high degree of sperm capacitation and maximises IVF efficiency in porcine. In conclusion, adjusting the HCO_3_^−^ concentration of the medium during in vitro epididymal sperm capacitation and fertilisation allowed us to increase the monospermy efficiency of these in vitro processes in porcine, which are currently suboptimal. In addition, a concentration of 15 mmol/L HCO_3_^−^ in the capacitating medium is also sufficient to activate the sperm capacitation cascade and induce the slower progression of the sAC/cAMP/PKA pathway. A concentration of 15 mmol/L produces a sufficient level of activation of sperm motility, pH_i_ increase and Tyr-P, maintaining high levels of fertilizing ability and increased rates of monospermy.

## Additional files

Additional file 1: Effect of HCO_3_^−^ on boar epididymal sperm intracellular pH (pH_i_). **A**) Calibration of the system in which fluorescence emission ratio 490/440 nm was detected when stained spermatozoa with BCECF-AM were exposed to different extracellular pH (pH_e_) in the presence of 0.1% Triton X-100 and excited both at 490 and 440 nm. The regression line for pH_e_ vs. the 490/440 nm ratio was obtained (y = 3.048x + 15.534) and pH_i_ of sperm cells was estimated from that regression line. **B**) Detection of fluorescence (arbitrary units) for 300 s when BCECF-AM stained spermatozoa were excited both at 490 (dark grey line) and 440 nm (light grey line) and the ratio 490/440 nm (black line). (TIF 7 kb)

Additional file 2: Cluster centers for each CASA motility parameter used to classify each spermatozoa of the study according to their degree of similarity. Cluster 1: spermatozoa with the poorest motility (lowest values in all CASA parameters); Cluster 2: spermatozoa with the most linear trajectory (high values of VSL, LIN, STR and WOB); Cluster 3: spermatozoa with the most curvilinear trajectory (high values of VCL and ALH and low values of VSL, LIN and STR). VCL, μm/s: curvilinear velocity; VSL, μm/s: straight-line velocity; VAP, μm/s: average path velocity; LIN, %: linearity of the curvilinear trajectory; STR, %: straightness; WOB, %: Wobble (VAP/VCL); ALH, μm: amplitude of lateral head displacement; BCF, Hz: beat cross-frequency. (PDF 28 kb)

Additional file 3: Patterns of immunolocation of protein tyrosine phosphorylation (Tyr-P) on boar epididymal sperm. The Tyr-P location of spermatozoa proteins were classified and grouped into 4 different categories according to the four patterns described by Luño et al. [28]: i) Pattern I (low capacitation status), which included spermatozoa without fluorescence **(A)** or with phosphorylated acrosome **(B)** or tail **(C)** or acrosome and tail **(D)**; ii) Pattern II (medium capacitation status), which included spermatozoa with fluorescence in the equatorial subsegment with **(E)** or without **(F)** the presence of signal in the flagellum; iii) Pattern III (high capacitation status), which included spermatozoa with signal in the equatorial subsegment and acrosome area and with **(G)** or without **(H)** the presence of signal in the flagellum; Pattern IV (motility activation status), which included those spermatozoa with a signal in the flagellum regardless of other locations **(I)**. (TIF 12 kb)

Additional file 4: **A**) Sperm plasma membrane integrity evaluated with eosin-nigrosin staining as a reflection of spermatozoa viability. Spermatozoa viability was classified as i) viable: membrane-intact spermatozoa, impermeable to eosin-nigrosin staining (black arrows) or ii) non-viable: membrane-altered spermatozoa, permeable to eosin-nigrosin staining (black asterisk). **B**) Effect of HCO_3_^−^ concentration on sperm viability. Epididymal sperm samples were incubated at 38.5 °C for 1 and 60 min in a capacitating medium (TALP) with different concentrations of HCO_3_^−^ (0 mmol/L, 5 mmol/L, 15 mmol/L and 25 mmol/L) and in non-capacitating medium (NCAP). Sperm plasma membrane integrity was evaluated using the eosin-nigrosin staining technique. Seven replicates were performed, in which 200 spermatozoa per experimental group were classified as viable (membrane-intact) or non-viable (membrane-altered). Results are shown as mean ± SEM. Data were analysed by one-way ANOVA and a Tukey test of multiple comparisons. Different letters (a-b) indicate statistically significant differences between groups in the same time of incubation (*P* < 0.05). (TIF 9 kb)

Additional file 5: Effect of HCO_3_^−^ concentration on acrosome reaction (AR). Epididymal sperm samples were incubated at 38.5 °C for 1 and 60 min in a capacitating medium (TALP) with different concentrations of HCO_3_^−^ (0 mmol/L, 5 mmol/L, 15 mmol/L and 25 mmol/L) and in non-capacitating medium (NCAP). AR was assessed by staining with 1% FITC-conjugated peanut agglutinin from *Arachis hypogaea* (PNA-FITC L7381, Sigma-Aldrich®, Madrid, Spain) 20 μg/mL. Samples were fixed at 1% formol-saline, smeared onto glass slides and coverslips and analysed by epifluorescence microscopy (blue filter, BP 480/40; emission BP 527/30; Leica® DM4000 B LED, USA) at × 400. Four replicates were performed, in which 200 spermatozoa per sample were analysed, classifying the sperm in reacted or non-reacted (with or without fluorescence in the acrosomal region, respectively). Results are shown as mean ± SEM. Data were analysed by one-way ANOVA and a Tukey test of multiple comparisons. Different letters (a-b) in the same time of incubation indicate statistically significant differences (*P* < 0.05). (TIF 4 kb)

## Abbreviations

Adcy10: Atypical soluble adenylyl cyclase Adcy10 aka sAC
ALH: Amplitude of lateral head displacement
AR: Acrosome reaction
BCF: Beat cross-frequency
BSA: Bovine serum albumin
CASA: Computer-assisted spermatozoa motility analysis
COCs: Cumulus-oocytes complexes
HCO_3_^−^: Bicarbonate
IIF: Indirect immunofluorescence
IVF: In vitro fertilisation
LIN: Linearity of the curvilinear trajectory
Mon: Percentage of monospermy of penetrated oocytes
Mot: Percentage of total motile spermatozoa
MotPro: Motile progressive spermatozoa
NBC: Electrogenic Na^+^/HCO_3_^−^ co-transporter
NCAP: Non-capacitating control
PBS: Phosphate buffer saline
Pen: Percentage of penetrated oocytes
pH_e_: Extracellular pH
pH_i_: Intracellular pH
PKA: Protein kinase A
pPKAs: Phosphorylation of protein kinase A substrates
SEM: Standard error of the mean
SPZ/O: Number of spermatozoa penetrating each oocyte
SPZ/ZP: Number of sperm bound to ZP
STR: Straightness
TALP: Tyrode’s albumin lactate pyruvate media
Tyr-P: Tyrosine phosphorylation
VAP: Average path velocity
VCL: Curvilinear velocity
VSL: Straight line velocity
WOB: Wobble of the curvilinear trajectory
ZP: Zona pellucida
β-act: Anti-β-actin HRP
β-tub: Anti-β-tubulin
