# Supplementary figures and images for: Manipulation of bicarbonate concentration in sperm capacitation media improvesin vitro fertilisation output in porcine species
Source: J Anim Sci Biotechnol. 2019 Mar 11;10:19. doi: 10.1186/s40104-019-0324-y (PMC6410524; doi:10.1186/s40104-019-0324-y)

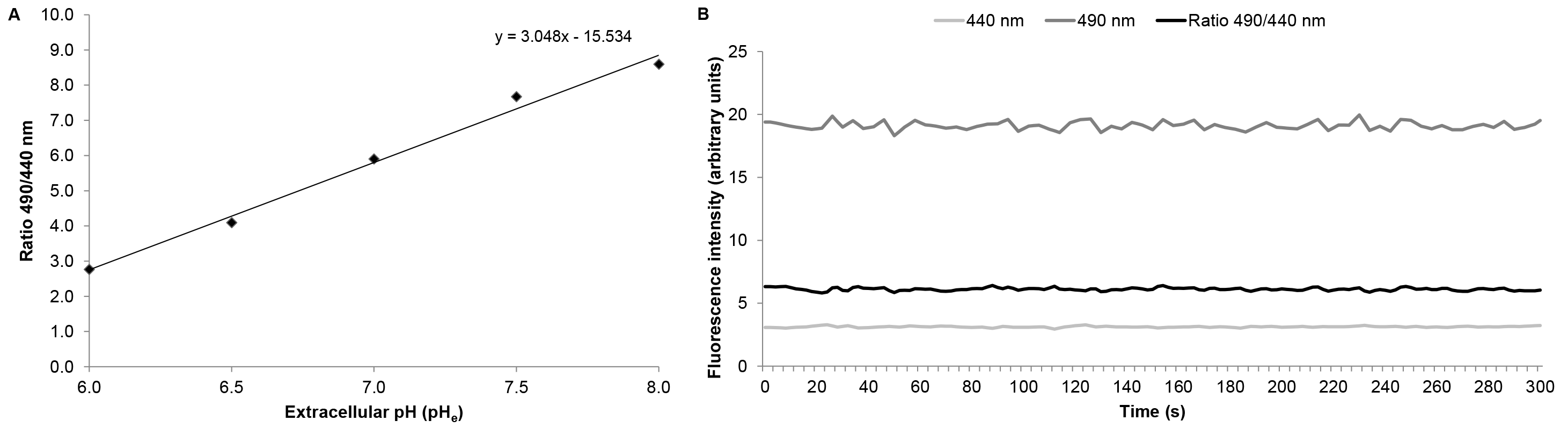

Supplement: Supplementary file 1 — Effect of HCO3− on boar epididymal sperm intracellular pH (pHi). A) Calibration of the system in which fluorescence emission ratio 490/440 nm was detected when stained spermatozoa with BCECF-AM were exposed to different extracellular pH (pHe) in the presence of 0.1% Triton X-100 and excited both at 490 and 440 nm. The regression line for pHe vs. the 490/440 nm ratio was obtained (y = 3.048x + 15.534) and pHi of sperm cells was estimated from that regression line. B) Detection of fluorescence (arbitrary units) for 300 s when BCECF-AM stained spermatozoa were excited both at 490 (dark grey line) and 440 nm (light grey line) and the ratio 490/440 nm (black line). (TIF 7 kb) [file 40104_2019_324_MOESM1_ESM.tif]

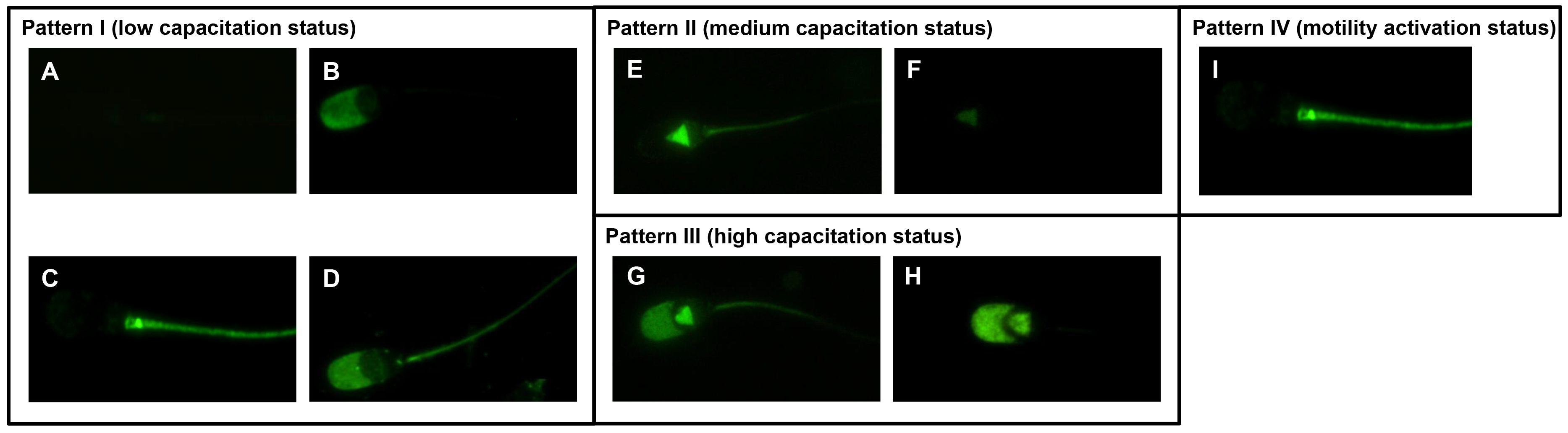

Supplement: Supplementary file 3 — Patterns of immunolocation of protein tyrosine phosphorylation (Tyr-P) on boar epididymal sperm. The Tyr-P location of spermatozoa proteins were classified and grouped into 4 different categories according to the four patterns described by Luño et al. [28]: i) Pattern I (low capacitation status), which included spermatozoa without fluorescence (A) or with phosphorylated acrosome (B) or tail (C) or acrosome and tail (D); ii) Pattern II (medium capacitation status), which included spermatozoa with fluorescence in the equatorial subsegment with (E) or without (F) the presence of signal in the flagellum; iii) Pattern III (high capacitation status), which included spermatozoa with signal in the equatorial subsegment and acrosome area and with (G) or without (H) the presence of signal in the flagellum; Pattern IV (motility activation status), which included those spermatozoa with a signal in the flagellum regardless of other locations (I). (TIF 12 kb) [file 40104_2019_324_MOESM3_ESM.tif]

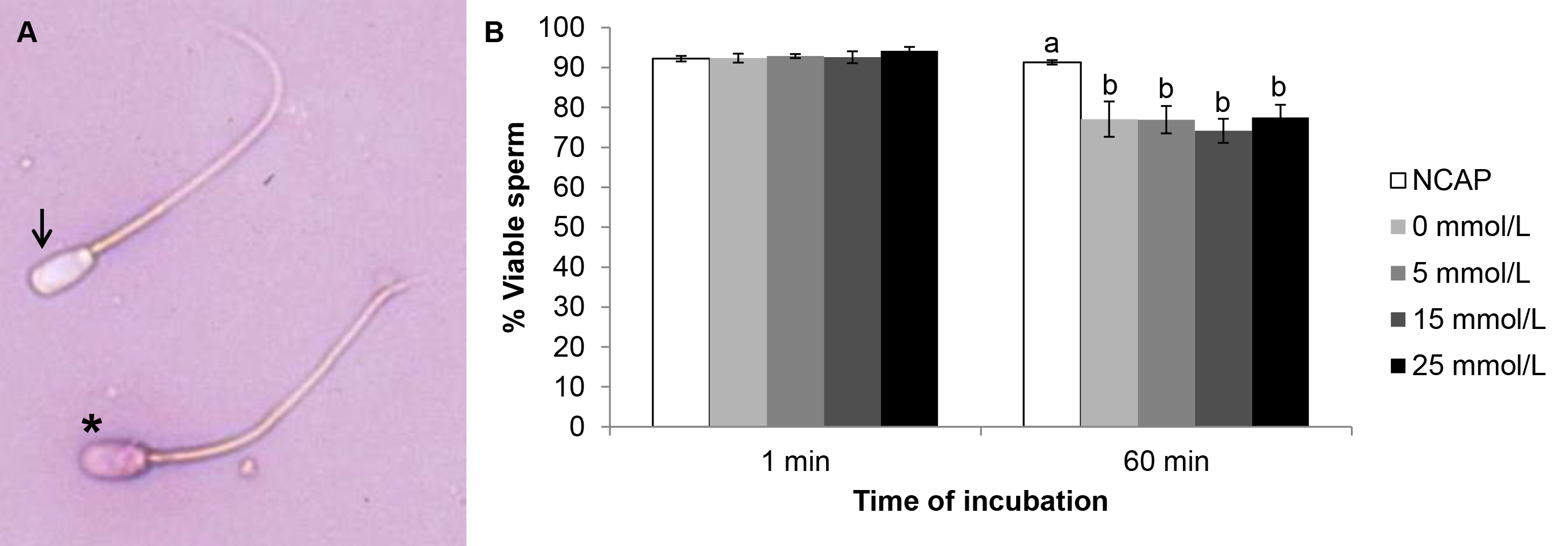

Supplement: Supplementary file 4 — A) Sperm plasma membrane integrity evaluated with eosin-nigrosin staining as a reflection of spermatozoa viability. Spermatozoa viability was classified as i) viable: membrane-intact spermatozoa, impermeable to eosin-nigrosin staining (black arrows) or ii) non-viable: membrane-altered spermatozoa, permeable to eosin-nigrosin staining (black asterisk). B) Effect of HCO3− concentration on sperm viability. Epididymal sperm samples were incubated at 38.5 °C for 1 and 60 min in a capacitating medium (TALP) with different concentrations of HCO3− (0 mmol/L, 5 mmol/L, 15 mmol/L and 25 mmol/L) and in non-capacitating medium (NCAP). Sperm plasma membrane integrity was evaluated using the eosin-nigrosin staining technique. Seven replicates were performed, in which 200 spermatozoa per experimental group were classified as viable (membrane-intact) or non-viable (membrane-altered). Results are shown as mean ± SEM. Data were analysed by one-way ANOVA and a Tukey test of multiple comparisons. Different letters (a-b) indicate statistically significant differences between groups in the same time of incubation (P < 0.05). (TIF 9 kb) [file 40104_2019_324_MOESM4_ESM.tif]

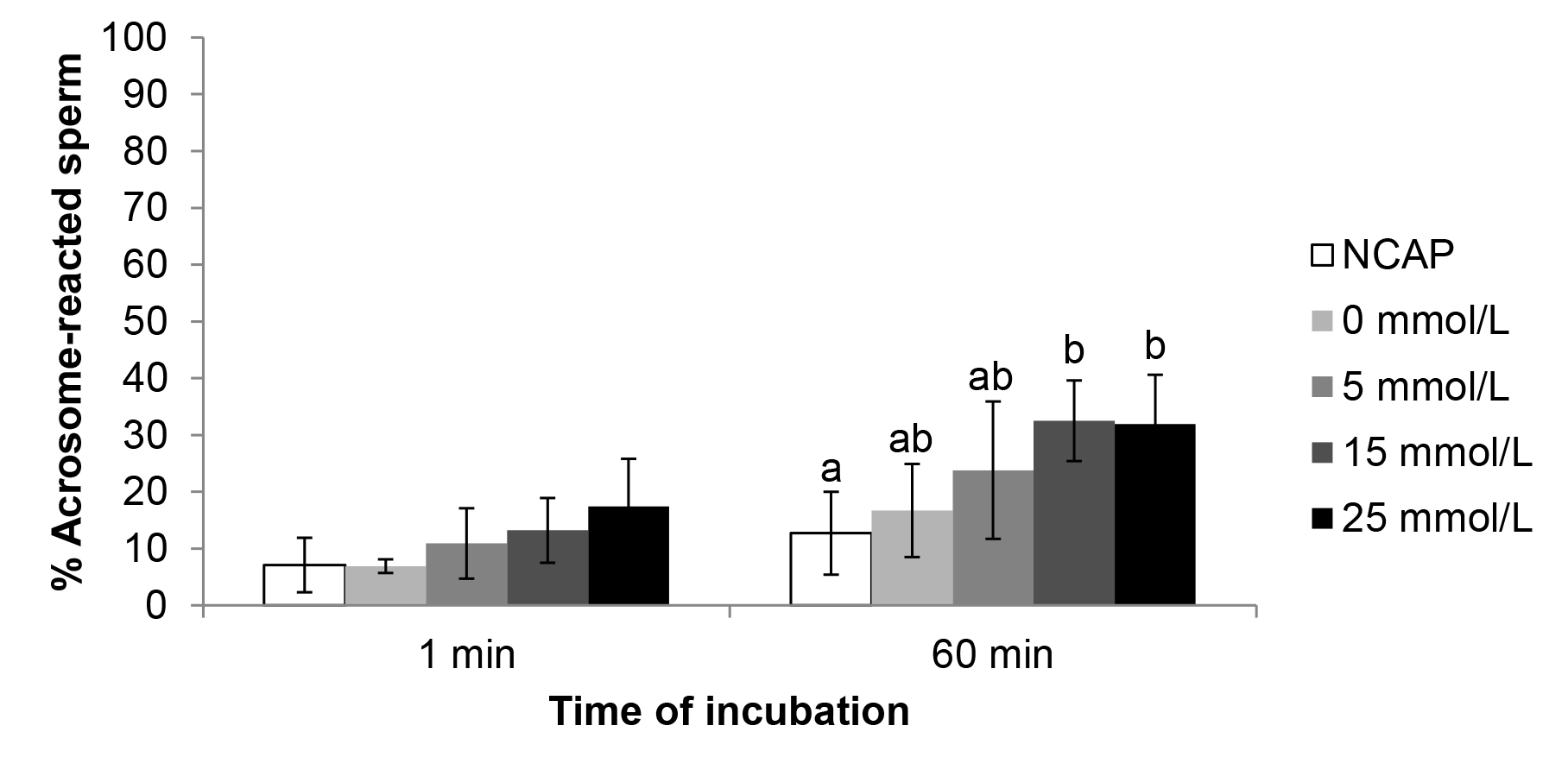

Supplement: Supplementary file 5 — Effect of HCO3− concentration on acrosome reaction (AR). Epididymal sperm samples were incubated at 38.5 °C for 1 and 60 min in a capacitating medium (TALP) with different concentrations of HCO3− (0 mmol/L, 5 mmol/L, 15 mmol/L and 25 mmol/L) and in non-capacitating medium (NCAP). AR was assessed by staining with 1% FITC-conjugated peanut agglutinin from Arachis hypogaea (PNA-FITC L7381, Sigma-Aldrich®, Madrid, Spain) 20 μg/mL. Samples were fixed at 1% formol-saline, smeared onto glass slides and coverslips and analysed by epifluorescence microscopy (blue filter, BP 480/40; emission BP 527/30; Leica® DM4000 B LED, USA) at × 400. Four replicates were performed, in which 200 spermatozoa per sample were analysed, classifying the sperm in reacted or non-reacted (with or without fluorescence in the acrosomal region, respectively). Results are shown as mean ± SEM. Data were analysed by one-way ANOVA and a Tukey test of multiple comparisons. Different letters (a-b) in the same time of incubation indicate statistically significant differences (P < 0.05). (TIF 4 kb) [file 40104_2019_324_MOESM5_ESM.tif]
