# Supplementary material for: Manipulation of bicarbonate concentration in sperm capacitation media improvesin vitro fertilisation output in porcine species
Source: J Anim Sci Biotechnol. 2019 Mar 11;10:19. doi: 10.1186/s40104-019-0324-y (PMC6410524; doi:10.1186/s40104-019-0324-y)
Supplement: Supplementary file 2 — Cluster centers for each CASA motility parameter used to classify each spermatozoa of the study according to their degree of similarity. Cluster 1: spermatozoa with the poorest motility (lowest values in all CASA parameters); Cluster 2: spermatozoa with the most linear trajectory (high values of VSL, LIN, STR and WOB); Cluster 3: spermatozoa with the most curvilinear trajectory (high values of VCL and ALH and low values of VSL, LIN and STR). VCL, μm/s: curvilinear velocity; VSL, μm/s: straight-line velocity; VAP, μm/s: average path velocity; LIN, %: linearity of the curvilinear trajectory; STR, %: straightness; WOB, %: Wobble (VAP/VCL); ALH, μm: amplitude of lateral head displacement; BCF, Hz: beat cross-frequency. (PDF 28 kb) [file 40104_2019_324_MOESM2_ESM.pdf]

	Cluster centres		
**Motility parameters**	**Cluster 1**	**Cluster 2**	**Cluster 3**
**VCL, µm/s**	104	154	267
**VSL, µm/s**	29	91	67
**VAP, µm/s**	51	103	104
**LIN, %**	30	61	25
**STR, %**	56	88	63
**WOB, %**	51	69	40
**ALH, µm**	2	3	6
**BCF, Hz**	15	21	18
